# How to Manage a Large Undifferentiated Lung Cancer Mass: A Case Report

**DOI:** 10.7759/cureus.75159

**Published:** 2024-12-05

**Authors:** Salman Arif, Akmal Irfan, Syed S Qadri

**Affiliations:** 1 Cardiothoracic Surgery, Hull University Teaching Hospitals NHS Trust, Cottingham, GBR

**Keywords:** adjuvant chemotherapy, neo-adjuvant chemotherapy, non-small cell carcinoma, right posterolateral thoracotomy, undifferentiated lung cancer

## Abstract

Non-small cell lung cancer is the most common type of lung cancer globally. An important subtype to discuss is undifferentiated carcinomas, which are variants of large cell carcinoma, and these can typically present with evidence of neuroendocrine differentiation.

The patient presented with a large mass in the right upper lobe extending into the middle lobe. It was attached to the pleura and involved the pericardium medially. The biopsy showed poorly differentiated carcinoma. However, pleural origin could not be ruled out. Hence, the patient was planned to have surgical excision followed by adjuvant chemotherapy. The surgical excision involved a video-assisted thoracic surgery (VATS) inspection followed by median sternotomy for medial dissection and then the excision was completed through right posterolateral thoracotomy. The patient recovered well postoperatively and went home on Day 7. The patient is doing well at four months follow-up.

It is interesting to know how a large tumour can be managed when the origin of the tumour is not certain and neoadjuvant therapy cannot be utilized to downsize the tumour.

## Introduction

Managing undifferentiated carcinoma is challenging since no clear histopathology is available and a clear plan of surgery with or without adjuvant chemotherapy vs. neoadjuvant therapy is difficult to make. In this case, there was suspicion that the tumour was radiologically arising from the pleura, which made it more challenging to find the best treatment plan. The location and size of the tumour made it interesting to see how it was completely resected surgically.

Non-small cell lung cancer encompasses approximately 85% of total lung cancer cases worldwide with several subtypes [1]. An important subtype to discuss is undifferentiated carcinomas, which are variants of large cell carcinoma, and these can typically present with evidence of neuroendocrine differentiation [2]. These carcinomas are highly aggressive and thus conventional neoadjuvant therapy is poorly indicated in these tumours because delaying surgical intervention decreases the prognosis for the patient.

## Case presentation

A 58-year-old female presented with a three-month history of a mild dry cough, which was worse on lying down. The patient often needed to clear her throat and denied any dysphagia, reflux, chest tightness, or a wheeze.

**Figure 1:**
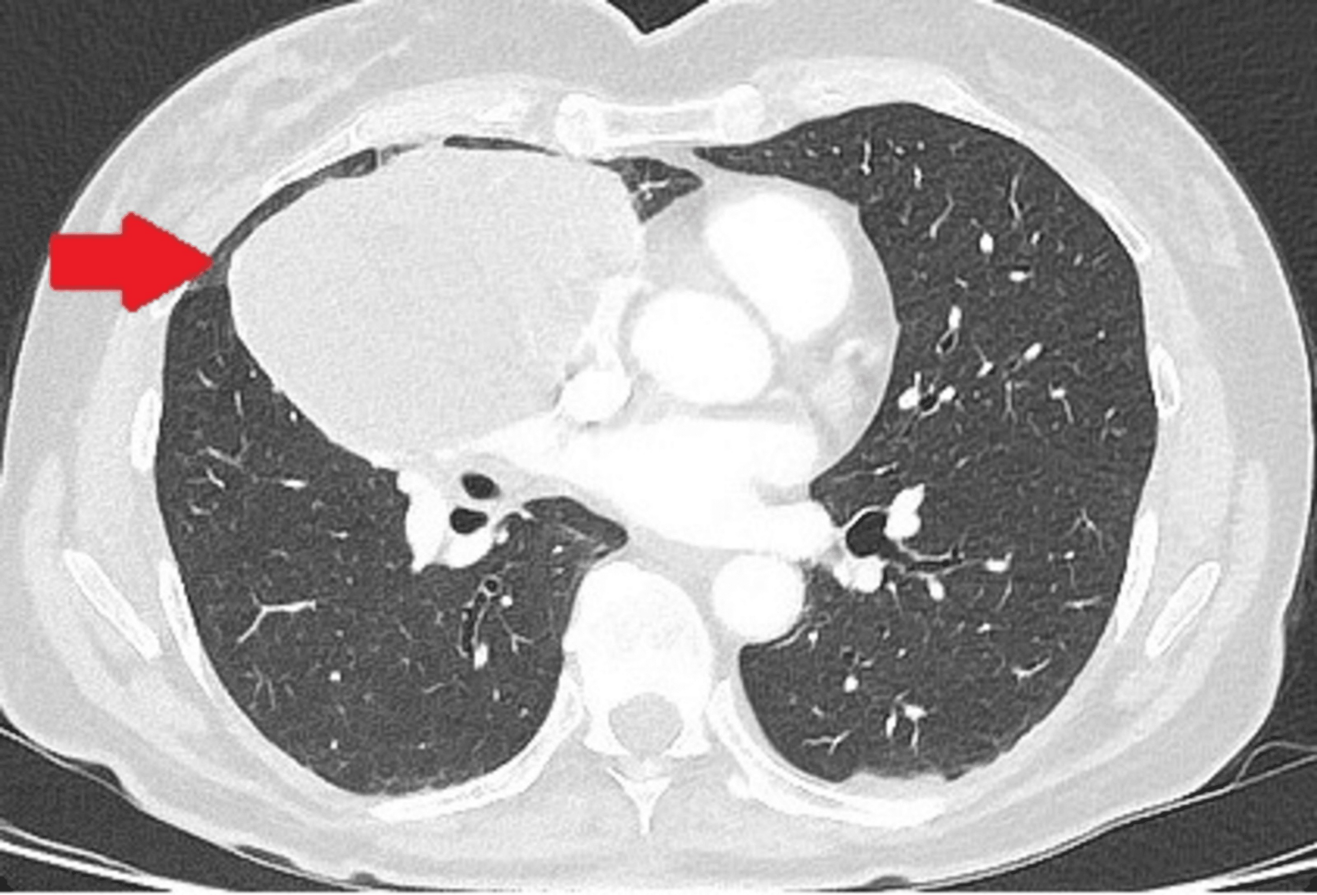
Preoperative CT scan The cross-sectional view of the CT scan shows a large mass in the right chest.

**Figure 2:**
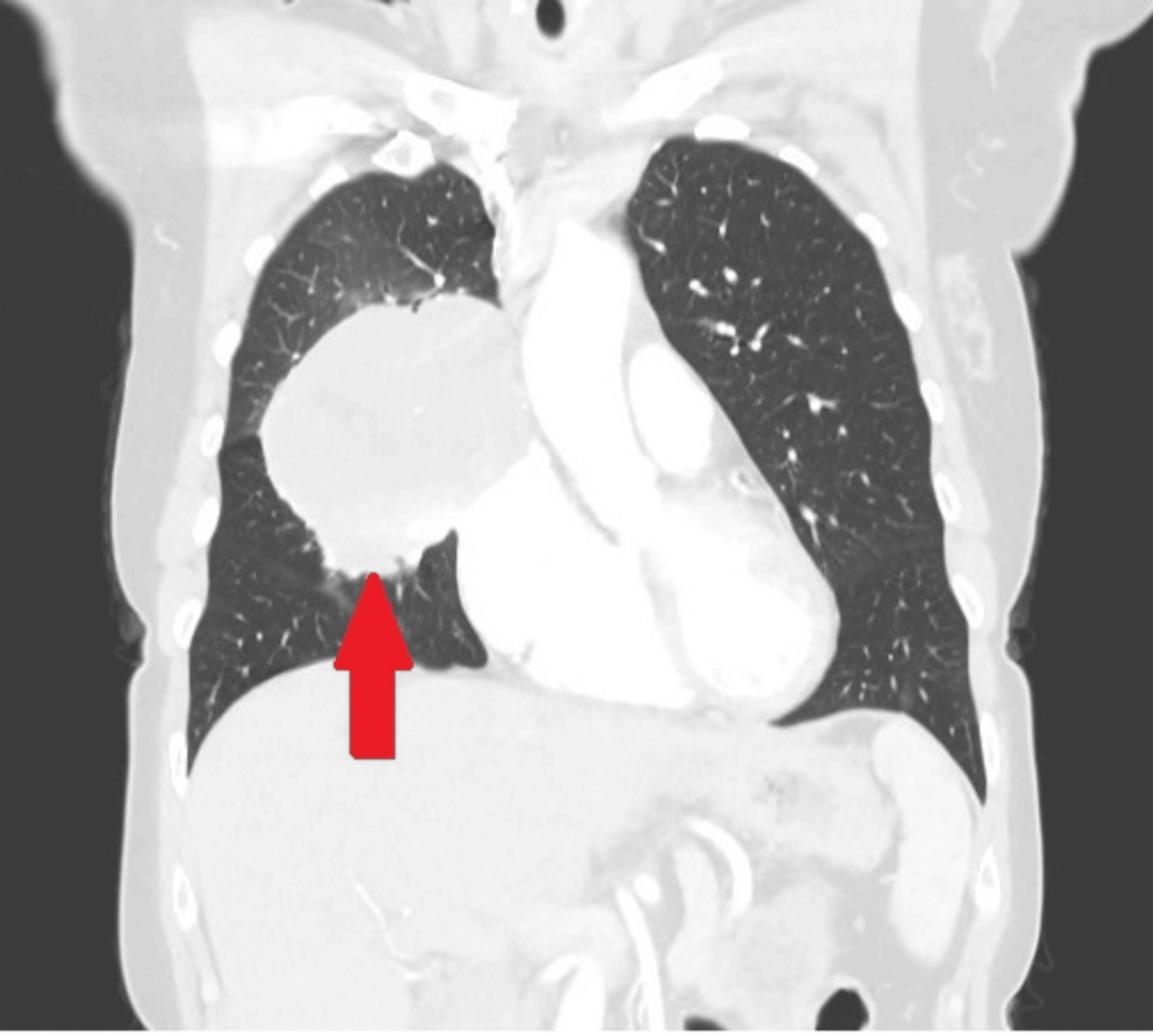
Preoperative CT scan (coronal section) The coronal sections of the CT scan show a large mass in the right upper and middle lobes and its attachment with the mediastinum.

A computed tomography (CT) scan detected a smooth and large 9 cm mass that predominantly occupied the upper and middle lobes of the right lung (Figure 1) (Figure 2). The mass was attached to the pleura; hence, there was suspicion that it could be pleural fibroma. Hence, a positron emission spectroscopy (PET) scan was done, which revealed the mass had a heterogeneous intense tracer activity, so the initial differential diagnosis was a PET-positive undifferentiated carcinoma of the lung. Neoadjuvant treatment was not considered, as there was a possibility that the tumour could be pleural-based. Therefore, the patient was referred for the surgical excision.

The surgical procedure conducted was a right video-assisted thoracic surgery (VATS) approach to rule out any pleural metastases and assess if it was resectable from the posterolateral thoracotomy approach alone. Once confirmed that there was no pleural involvement the surgeons proceeded to median sternotomy. The mediastinal aspect of the mass was mobilised from the pericardium and the phrenic nerve was sacrificed to achieve complete excision. However, it was deemed suitable that the bilobectomy be carried out from the posterolateral thoracotomy approach, as it was difficult to dissect the hilum from the median sternotomy due to the size and position of the tumour. The sternum was closed with six wires and the patient was turned to approach the hilum from the right posterolateral thoracotomy. The mass was further mobilised and an upper bilobectomy was done. The pericardium was repaired with a bovine pericardial patch. Two chest drains were inserted, and the thoracotomy closed in layers. The patient recovered well postoperatively and went home on Day 7.

The histology confirmed complete excision of the mass and post-operative imaging showed complete clearance of the lymph nodes. The tumour was confirmed poorly differentiated non-small cell lung cancer. Through immunochemical analysis, the tumour cells were positive for pan-cytokeratin, CAM 5.2, focally positive for D2 40 and few cells were positive for CD56 and chromogranin. Interestingly, it did have PD-L1 markers; however, this was only raised 1-2%. The tumour was negative for CD34 and its size was 95 mm. On further investigation, the tumour focally infiltrated the lung parenchyma and pleura and extended to the overlying fat present. It is composed of polygonal cells with moderate to markedly pleomorphic vesicular to hyperchromatic nuclei and eosinophilic cytoplasm. It was negative for EGFR, BRAF, KRAS, ALK, p40 and p63. Collectively, the lung specimen showed a poorly differentiated carcinoma with focal equivocal neuroendocrine differentiation and TNM was pT4N0. The patient received adjuvant chemotherapy using vinorelbine and carboplatin. On four months follow-up, the patient is doing well.

## Discussion

Due to the difficulty of distinguishing between small cell carcinomas and non-small cell lung cancers, in which this case had an undifferentiated carcinoma, it is imperative to analyse tissue on a histopathological basis to ensure appropriate treatment can be delivered.

When initially trying to distinguish between small cell carcinomas and non-small cell carcinomas with evidence of neuroendocrine differentiation of pulmonary origin, it is important to consider tumour markers such as chromogranin, CD56, p40 and p53. On histological analysis, the negative results of p40 and p53 tumour markers in this case suggest that this lesion is more fitting with that of non-small cell cancer as opposed to small cell lung cancer [3].

In the early stages of undifferentiated carcinomas with evidence of neuroendocrine differentiation, surgery and complementary adjuvant chemotherapy, including etoposide and platinum are often the gold standard intervention to remove the cancer to prevent cancer resurgence [4]. In this study for advanced neuroendocrine lung carcinomas, chemotherapy alone offered statistically significant results in survival time compared to radiotherapy alone. However, a combination of both chemotherapy and radiotherapy did not confer a statistically significant improvement over just chemotherapy in terms of survival times.

In another study, there is approximately a 4-month increase of 38 months compared to 34 months in medial survival time in patients receiving surgery and adjuvant chemotherapy as opposed to just surgery alone [2]. Furthermore, another study suggested that the 5-year survival rate was 87.5% when using adjuvant chemotherapy alongside surgery but significantly decreased to 58.5% if surgery was used in isolation [5]. Here, this further adds to the narrative that the current best treatment regimen for undifferentiated carcinomas of the lung remains to be surgery with adjuvant chemotherapy.

Since undifferentiated carcinomas with neuroendocrine differentiation fit under the umbrella of non-small cell lung cancer, it is thought that molecular targeted therapy could suffice. This is because of the concept of driver mutation where the cancer proliferation is highly dependent on one oncogene thus making it easier for targeted therapy. However, instances of driver mutation are rare in large-cell neuroendocrine carcinomas, suggesting targeted therapy can be a challenge, especially in cases similar to this. Immunotherapies have also been devised for non-small cell lung cancer, especially regarding the expression of PD-L1. This represents a checkpoint that can be a target to prevent tumour proliferation. However, this ligand is rarely positive in findings of a large cell carcinoma and especially in the context of this case, pd-L1 was present in only 1-2% of cells, which renders this form of immunotherapy potentially ineffective in the treatment of this undifferentiated carcinoma [6].

## Conclusions

This is an interesting case, as the origin of the tumour was not clear in the beginning. Also, the size and position of the tumour made it an interesting case, as we utilised VATS, median sternotomy and right posterolateral thoracotomy approaches to achieve complete excision.

Although several treatment regimens exist for undifferentiated carcinomas, it is important to consider the genetic profiling of these tumours, as this can significantly impact what therapeutic intervention is best indicated. From the evidence gathered, it can be suggested that the combination of radical surgery and adjuvant chemotherapy remains the best solution in treating undifferentiated carcinomas of the lung with evidence of neuroendocrine differentiation. The aggressive nature, that these carcinomas tend to display, can render traditional treatment of non-small cell lung cancer potentially ineffective such as the use of neoadjuvant therapy over immediate surgical intervention.

## References

[1] Hu J, Zhang L, Xia H (2023). Tumor microenvironment remodeling after neoadjuvant immunotherapy in non-small cell lung cancer revealed by single-cell RNA sequencing. Genome Med.

[2] Tai Q, Zhang L, Hu X (2020). Clinical characteristics and treatments of large cell lung carcinoma: a retrospective study using SEER data. Transl Cancer Res.

[3] Kriegsmann K, Zgorzelski C, Muley T (2021). Role of Synaptophysin, Chromogranin and CD56 in adenocarcinoma and squamous cell carcinoma of the lung lacking morphological features of neuroendocrine differentiation: a retrospective large-scale study on 1170 tissue samples. BMC Cancer.

[4] Xia L, Wang L, Zhou Z, Han S (2022). Treatment outcome and prognostic analysis of advanced large cell neuroendocrine carcinoma of the lung. Sci Rep.

[5] Saji H, Tsuboi M, Matsubayashi J (2010). Clinical response of large cell neuroendocrine carcinoma of the lung to perioperative adjuvant chemotherapy. Anticancer Drugs.

[6] Ferrara MG, Stefani A, Simbolo M (2021). Large cell neuro-endocrine carcinoma of the lung: current treatment options and potential future opportunities. Front Oncol.

